# Nomogram for predicting lymph node metastasis in patients with ovarian cancer using ultrasonography: a multicenter retrospective study

**DOI:** 10.1186/s12885-023-11624-5

**Published:** 2023-11-17

**Authors:** Yaqin Yang, Xuewei Ye, Binqian Zhou, Yang Liu, Mei Feng, Wenzhi Lv, Dan Lu, Xinwu Cui, Jianxin Liu

**Affiliations:** 1 Department of Ultrasound, Tongji Medical College, The Central Hospital of Wuhan, Huazhong University of Science and Technology, Wuhan, China; 2 Department of Artificial Intelligence, Julei Technology Company, Wuhan, 430030 China; 3 Department of Ultrasound, Zhongnan Hospital of Wuhan University, Wuhan, China; 4 Department of Medical Ultrasound, Tongji Hospital, Tongji Medical College, Huazhong University of Science and Technology, Wuhan, China

**Keywords:** Ovarian cancer, Lymph node Metastasis, Ultrasound, Prediction model

## Abstract

**Background:**

Ovarian cancer is a common cancer among women globally, and the assessment of lymph node metastasis plays a crucial role in the treatment of this malignancy. The primary objective of our study was to identify the risk factors associated with lymph node metastasis in patients with ovarian cancer and develop a predictive model to aid in the selection of the appropriate surgical procedure and treatment strategy.

**Methods:**

We conducted a retrospective analysis of data from patients with ovarian cancer across three different medical centers between April 2014 and August 2022. Logistic regression analysis was employed to establish a prediction model for lymph node metastasis in patients with ovarian cancer. We evaluated the performance of the model using receiver operating characteristic (ROC) curves, calibration plots, and decision analysis curves.

**Results:**

Our analysis revealed that among the 368 patients in the training set, 101 patients (27.4%) had undergone lymph node metastasis. Maximum tumor diameter, multifocal tumor, and Ki67 level were identified as independent risk factors for lymph node metastasis. The area under the curve (AUC) of the ROC curve in the training set was 0.837 (95% confidence interval [CI]: 0.792–0.881); in the validation set this value was 0.814 (95% CI: 0.744–0.884). Calibration plots and decision analysis curves revealed good calibration and clinical application value.

**Conclusions:**

We successfully developed a model for predicting lymph node metastasis in patients with ovarian cancer, based on ultrasound examination results and clinical data. Our model accurately identified patients at high risk of lymph node metastasis and may guide the selection of appropriate treatment strategies. This model has the potential to significantly enhance the precision and efficacy of clinical management in patients with ovarian cancer.

**Supplementary Information:**

The online version contains supplementary material available at 10.1186/s12885-023-11624-5.

## Background

Ovarian cancer is one of the most common cancers in women worldwide, with approximately 313,000 new cases and 207,000 deaths in 2020, making it the third leading cause of death from gynecological malignancies [1]. Like most gynecological malignancies, the lymphatic system provides the primary route for ovarian cancer metastasis; the incidence of lymph node metastasis is lower in early- than in late-stage disease [2]. Lymph node status significantly affects the survival of patients with ovarian cancer and is an important factor in the International Federation of Gynecology and Obstetrics (FIGO) ovarian cancer staging system [3]. Ovarian cancer with lymph node metastasis is usually classified as stage III or higher and has a poorer prognosis. Lymph node dissection is an effective method of identifying lymph node metastasis and can therefore influence disease staging and prognosis, and guide treatment [4]. However, lymph node dissection in patients with ovarian cancer remains controversial, as it increases surgical duration and risk and may cause serious postoperative complications such as lower limb lymphedema and pelvic lymphatic cysts with accompanying infections [5]. It has been reported that 14.2% of patients with early-stage and 44–53% of patients with late-stage ovarian cancer may have lymph node metastasis [6]. Therefore, although determining lymph node status is important for ovarian cancer staging, not all patients require lymph node dissection.

Over the past few decades, numerous efforts have been made to develop accurate preoperative and intraoperative methods for identifying lymph node metastasis. Several models based on computed tomography (CT) or magnetic resonance imaging (MRI) have demonstrated significant predictive value [7]. However, CT does not provide sufficient accuracy for the prediction of lymph node metastasis, with a sensitivity of 48–80% [8]. MRI has a similar efficacy, with the additional disadvantage of increased examination costs. Positron emission tomography-CT can improve the staging accuracy, but has a high false positive rate [8]. Ultrasonography is the first-line imaging method for diagnosing ovarian masses with a high level of accuracy [9]. Furthermore, it is an inexpensive and safe procedure that does not pose a radiation risk to patients and is relatively easy to perform. Despite these advantages, further research is necessary to fully understand its diagnostic capabilities. The purpose of our study was to identify risk factors for lymph node metastasis in patients with ovarian cancer using ultrasound examination, in order to guide selection of the appropriate surgical procedure and treatment strategy.

## Methods

### Patients

Patients with ovarian cancer diagnosed between April 2014 and August 2022 at Wuhan Central Hospital, Zhongnan Hospital of Wuhan University, and Hubei Cancer Hospital were selected for this study. All patients underwent ovarian cancer resection and lymph node dissection. The inclusion criteria were as follows: (1) patients undergoing ultrasound examination owing to ovarian cancer; (2) a surgical and pathological diagnosis of primary ovarian cancer; and (3) a clear pathological report of lymph node status. The exclusion criteria were as follows: (1) patients receiving neoadjuvant radiotherapy or chemotherapy before surgery; (2) patients with concurrent or additional tumors; and (3) patients with incomplete clinical or ultrasound data before surgery. A total of 525 patients were included in the study. The study followed the principles of the Declaration of Helsinki and was approved by the Ethics Committee of Wuhan Central Hospital, Tongji Medical College, Huazhong University of Science and Technology. Due to the retrospective nature of the study, the requirement for informed consent was waived.

### Ultrasound examination

All patients underwent transvaginal ultrasound evaluation conducted by experienced sonographers using standardized examination techniques, as previously described [10]. If deemed necessary, transabdominal ultrasound was performed as a supplementary examination. All ultrasound examinations were performed using high-end ultrasound equipment. Transvaginal probes varied in frequency between 5.0 and 9.0 MHz, while transabdominal probes varied in frequency between 3.5 and 5.0 MHz.

### Study variables

Included study variables were the patient’s medical history (age, pregnancy history, reproductive history, age of menarche, menopause, and irregular bleeding), laboratory test results (detection of cancer antigen [CA]125, CA153, CA199, CA724, alpha-fetoprotein, carcinoembryonic antigen, squamous cell carcinoma antigen, human epididymis protein 4, and D-dimer; red blood cell, white blood cell [WBC], platelet, neutrophil, and lymphocyte counts; and calculation of the neutrophil-lymphocyte ratio), pathological examination data (histological type, FIGO stage, progesterone receptor, and Ki67), and ultrasound examination data (maximum tumor diameter, multifocal tumor, laterality, shape, echo, calcification, rear echo, intimal thickness, and presence of uterine fibroids and adenomyosis).

### Statistical analysis

Data analysis was performed using SPSS software version 26.0 (IBM Corp., Armonk, NY, USA), R software version 4.2.2, and GraphPad Prism version 9 (GraphPad Software, Inc., San Diego, CA, USA). Quantitative data are presented as the mean ± standard deviation, and qualitative data are presented as numbers (rates). Differences between groups were analyzed using Student’s t-tests and chi-square tests. Univariate binary logistic regression was used to analyze the risk factors for lymph node metastasis. Variables with *p* < 0.1 in the univariate analysis were included in the multivariate analysis. Multivariate analysis was performed using forward stepwise regression, and receiver operating characteristic (ROC) curves and nomograms were created based on the results. Decision curve analysis and calibration curves were used to evaluate the predictive performance of the nomograms. A p-value of less than 0.05 was considered statistically significant.

## Results

### Baseline information of patients

**Fig. 1:**
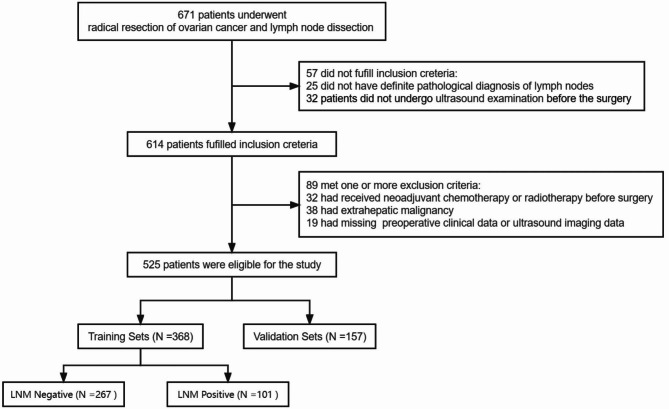
Flow chart including patient enrollment, inclusion, and exclusion criteria

We divided 525 patients into a training set (n = 368) and a validation set (n = 157) using a randomization method; the study flowchart is presented in Fig. 1. Among the study variables, age (53.92 ± 10.90 vs. 51.73 ± 11.30 years, *p* = 0.041) and maximum tumor diameter (89.53 ± 50.74 vs. 99.89 ± 52.99 mm, *p* = 0.035) differed significantly between training and validation sets; no other statistically significant differences were observed (Table S1 and S2). In the training set of 368 patients, 101 (27.4%) had lymph node metastasis (LNM positive group), and 267 (72.6%) did not (LNM negative group) (Table 1). Among the 368 patients in the training set, 237 (64%) had serous ovarian carcinoma, 15 (4.08%) had mucinous ovarian carcinoma, 22 (5.98%) had endometrioid ovarian carcinoma, and 80 (21.74%) had ovarian clear cell carcinoma. There were significant differences in the histological type, FIGO stage, and laterality of the tumor between the two groups. In addition, the expression levels of Ki67 in the LNM negative group were significantly lower than those in the LNM positive group (45.82 ± 28.04 vs. 66.68 ± 19.40, *p* < 0.001). In addition, there was a lower incidence of multifocal tumors in the LNM negative group (81 patients [30.3%]) than in the LNM positive group (77 patients [76.23%], *p* < 0.001) (Table 2), and the maximum tumor diameter in the LNM negative group was significantly smaller than that in the LNM positive group (81.67 ± 49.22 vs. 110.31 ± 49.02, *p* < 0.001). CT and MR findings also reflected similar differences in maximum tumor diameter and imaging staging between the two groups (Table S3). There were no significant differences in the other study variables between the two groups.

**Table 1:** Baseline clinical data of patients in the training set

Variables	All patients (N = 368)	LNM Negative (N = 267)	LNM Positive (N = 101)	*P* values
Age (year)	53.92 ± 10.90	53.77 ± 11.48	54.33 ± 9.21	0.663
Pregnancy history	2.95 ± 1.77	2.99 ± 1.84	2.86 ± 1.55	0.535
Reproductive history	1.59 ± 1.02	1.58 ± 1.06	1.60 ± 0.90	0.900
Menarche age (year)	13.39 ± 1.70	13.36 ± 1.74	13.47 ± 1.57	0.593
Menopause	245(66.58)	176(65.92)	69(68.32)	0.663
Irregular bleeding	14(3.80)	9(3.37)	5(4.95)	0.488
Histological type				0.002
SOC	237(64.40)	156(58.43)	81(80.20)	
MOC	15(4.08)	14(5.24)	1(0.10)	
EEOC	22(5.98)	20(7.49)	2(1.98)	
OCCC	80(21.74))	66(24.72)	14(13.86)	
Others	14(3.80)	11(4.12)	3(2.97)	
FIGO stage				< 0.001
I	109(29.62)	101(37.83)	8(7.92)	
II	55(14.95)	49(18.35)	6(5.94)	
III	163(44.29)	98(34.70)	65(64.36)	
IV	41(11.14)	19(7.12)	22(21.78)	
PR (+)	111(30.16)	82(30.71)	29(28.71)	
Ki67 (%)	51.54 ± 27.56	45.82 ± 28.04	66.68 ± 19.40	< 0.001
CA125 (U/mL)	863.98 ± 15.4.24	895.78 ± 1586.59	771.30 ± 1237.38	0.519
CA153 (U/mL)	54.78 ± 80.75	55.01 ± 81.66	54.16 ± 79.17	0.954
CA199 (U/mL)	812.87 ± 8483.40	1063.34 ± 9787.89	61.47 ± 171.43	0.377
CA724 (IU/mL)	46.76 ± 113.23	40.65 ± 62.35	63.99 ± 195.89	0.270
AFP (ng/mL)	13.03 ± 136.41	16.05 ± 156.17	3.32 ± 1.84	0.562
CEA (ng/mL)	5.79 ± 27.08	6.46 ± 30.12	3.70 ± 13.80	0.521
SCC (ng/mL)	0.82 ± 0.989	0.87 ± 1.11	0.65 ± 0.40	0.250
HE4 (pmol/L)	279.93 ± 326.42	307.14 ± 347.67	213.93 ± 260.24	0.129
D-dimer (ug/mL)	52.00 ± 346.13	57.39 ± 383.58	37.46 ± 215.75	0.631
RBC (x10^12^/L)	3.68 ± 0.63	3.67 ± 0.62	3.70 ± 0.67	0.683
WBC (x10^9^/L)	8.18 ± 16.64	8.72 ± 19.47	6.77 ± 3.00	0.318
Neutrophil (%)	70.82 ± 12.71	70.31 ± 13.08	72.14 ± 11.64	0.218
Lymphocyte (%)	20.04 ± 9.86	20.50 ± 9.82	18.86 ± 9.92	0.157
NLR	5.61 ± 6.57	5.30 ± 5.52	6.44 ± 8.70	0.137
PLT (x10^9^/L)	275.68 ± 112.81	281.79 ± 118.54	259.84 ± 95.08	0.097

Abbreviations: SOC, serous ovarian carcinoma; MOC, mucinous ovarian carcinoma; EEOC, endometrioid ovarian carcinoma; OCCC, ovarian clear cell carcinoma; FIGO, the international federation of gynecology and obstetrics; NLR, neutrophil-to-lymphocyte ratio. Data are presented as mean ± standard deviation or n (%)

**Table 2:** Baseline ultrasound data of patients in the training set

Variables	All patients (N = 368)	LNM Negative (N = 267)	LNM Positive (N = 101)	P values
Maximum tumor diameter (mm)	89.53 ± 50.74	81.67 ± 49.22	110.31 ± 49.02	< 0.001
Multifocal tumor	158(42.93)	81(30.33)	77(76.23)	< 0.001
Laterality				< 0.001
Left	127(34.51)	105(39.32)	22(21.78)	
Right	105(28.53)	84(31.46)	21(20.79)	
Bilateral	136(36.96)	78(29.21)	58(57.43)	
Shape, circle	189(51.36)	135(50.56)	54(53.46)	0.619
Homogeneous echoic	284(77.17)	205(76.78)	79(78.21)	0.769
Calcification	15(4.08)	10(3.75)	5(4.95)	0.602
Rear echo	117(31.79)	92(34.46)	25(24.75)	0.074
UF	131(35.60)	93(34.83)	38(37.62)	0.618
AM	23(6.25)	16(5.99)	7(6.93)	0.791
Intimal thickness (mm)	3.22 ± 4.24	3.29 ± 4.32	3.04 ± 4.00	0.681

Abbreviations: UF, uterine fibroids; AM, adenomyosis. Data are presented as mean ± standard deviation or n (%)

All patients underwent lymph node dissection, with a mean of 21.2 lymph nodes removed per patient (range: 2–53). Of the 101 patients who developed lymph node metastases in the training set, 48 (47.5%) had pelvic lymph node metastases, 13 (12.9%) had para-aortic lymph node metastases, and 40 (39.6%) had both pelvic and para-aortic lymph node metastases (Table 3).

**Table 3:** Lymph node metastasis in patients in the training and validation sets

Lymph node location	Training Set (LNM positive = 101)	Validation Set (LNM positive = 50)
Pelvic nodal metastase	48 (47.5)	24 (48.0)
Para-aortic nodal metastase	13 (12.9)	7 (14.0)
Pelvic and para-aortic nodal metastases	40 (39.6)	19 (38.0)

Data are presented as mean ± standard deviation or n (%)

### Univariate and multivariate regression analyses of risk factors for lymph node metastasis in ovarian cancer

To investigate the risk factors for lymph node metastasis in patients with ovarian cancer, we included the patients’ clinical and ultrasound data in a univariate logistic regression analysis (Table 4). The univariate regression analysis identified maximum tumor diameter, multifocal tumor, laterality, rear echo, histologic type, Ki67, and WBC as risk factors for lymph node metastasis. Variables with *p* < 0.1 in the univariate analysis were included in the multivariate analysis, which identified maximum tumor diameter (odds ratio [OR] = 1.012, 95% confidence interval [CI]: 1.006–1.019, *p* < 0.001), multifocal tumor (OR = 6.014, 95% CI: 3.164–11.431, *p* < 0.001), and Ki67 (OR = 1.023, 95% CI: 1.010–1.036, *p* < 0.001) as independent risk factors for lymph node metastasis.

**Table 4:** Univariate and multivariate Logistic regression analyses

Variables	Univariate Analysis			Multivariate Analysis		
	OR	95%CI	p	OR	95%CI	p
Maximum tumor diameter (mm)	1.011	1.006–1.016	< 0.001	1.012	1.006–1.019	< 0.001
Multifocal tumor	7.367	4.348–12.483	< 0.001	6.014	3.164–11.431	< 0.001
Laterality			< 0.001			
Right vs. Left	1.196	0.608–2.356	0.604			
Bilateral vs. Right	3.172	2.070–6.654	< 0.001			
Rear echo	0.594	0.347–1.018	0.058			
Histological type			0.003			
MOC vs. SOC	0.138	0.018–1.065	0.057			
EEOC vs. SOC	0.193	0.044–0.844	0.029			
OCCC vs. SOC	0.409	0.216–0.772	0.006			
Others vs. SOC	0.241	0.030–1.958	0.183			
Ki67 (%)	1.034	1.023–1.045	< 0.001	1.023	1.010–1.036	< 0.001
WBC (x10^9^/L)	0.942	0.879–1.010	0.092			

Abbreviations: OR, odds ratio; 95%CI, confidence interval

### Establishment and evaluation of nomogram

**Fig. 2:**
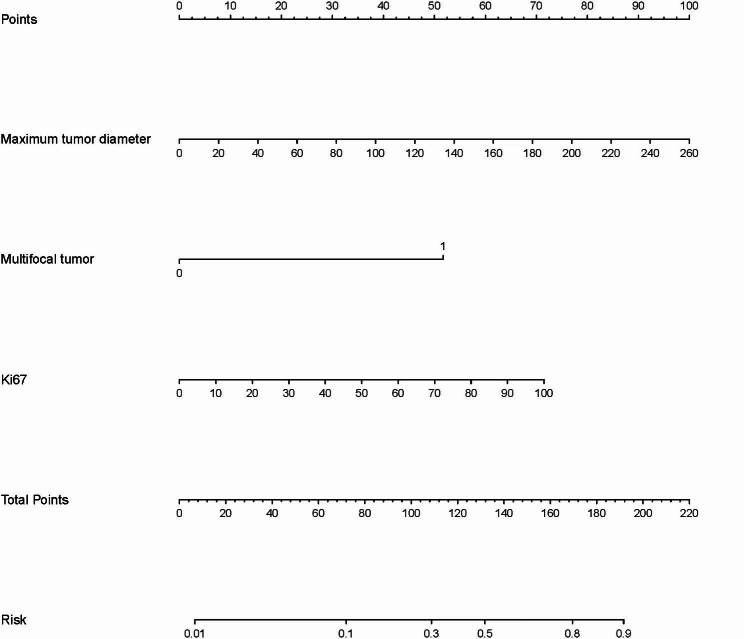
Nomogram for lymph node metastasis in ovarian cancer patients. A dynamic nomogram was also made (https://lnm-prediction-model.shinyapps.io/DynamicNomogramofLNM). Abbreviation: LNM, lymph node metastasis

**Fig. 3:**
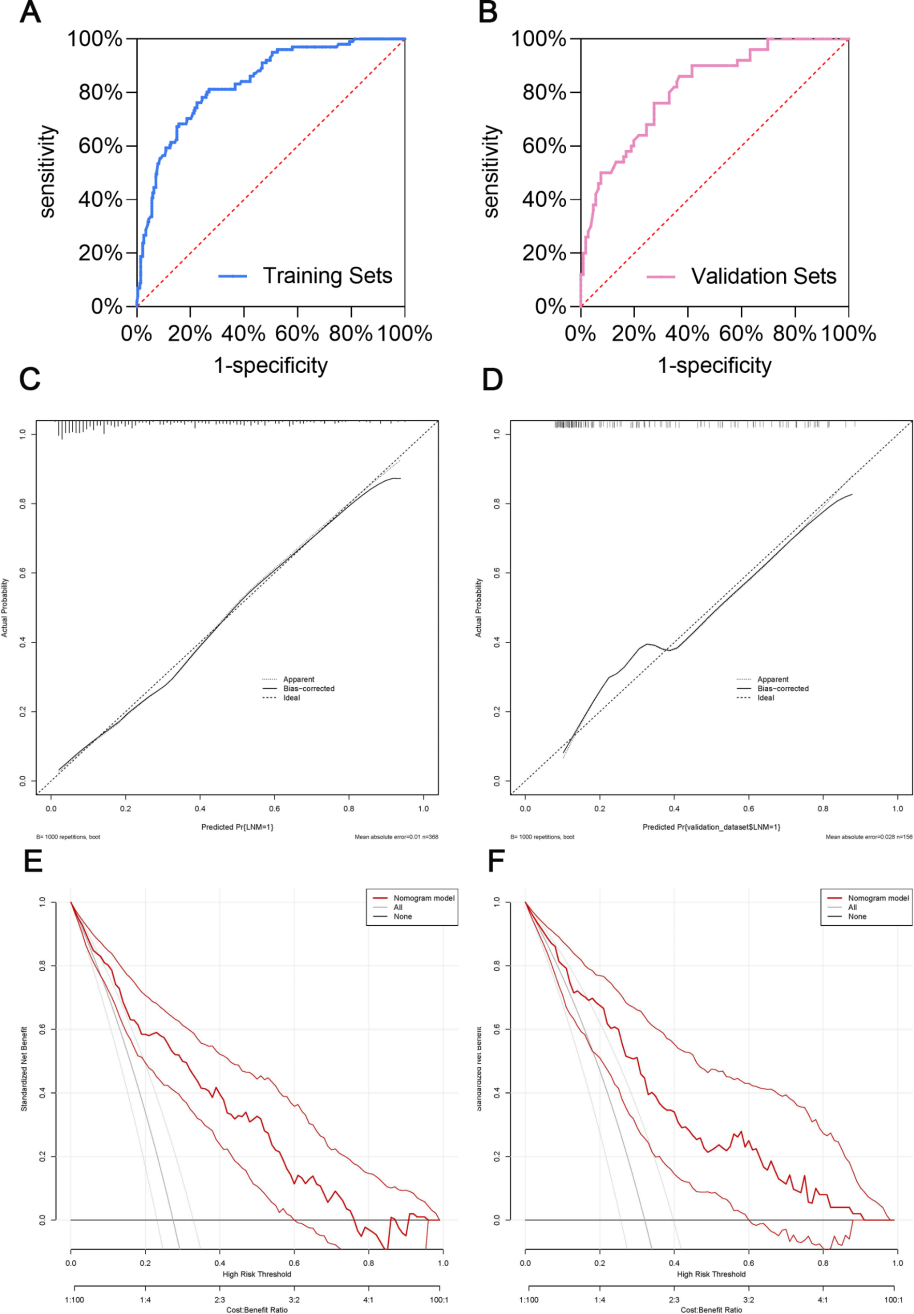
ROC curve, calibration plot, and decision analysis curve were used to evaluate the prediction model. (**A**) ROC curve for the training set. (**B**) ROC curve for the validation set. (**C**) calibration plot for the training set. (**D**) calibration plot for the validation set. (**E**) decision analysis curve for the training set. (**F**) decision analysis curve the validation set

Based on the results of the multivariate analysis, maximum tumor diameter, multifocal tumor, and Ki67 were included in the model to establish a nomogram (Fig. 2). Evaluation of the nomogram is presented in Fig. 3. The ROC curve was used to evaluate the performance of the model, with an area under the curve (AUC) of 0.837 (95% CI: 0.792–0.881) indicating that the model had good discrimination (Fig. 3A). In other words, the model had a high accuracy in distinguishing patients with and without lymph node metastasis. In addition, we used a calibration plot to evaluate the consistency between the predicted probability of lymph node metastasis and the actual occurrence of lymph node metastasis, which showed good calibration (Fig. 3C). Finally, we used a decision curve to evaluate the clinical utility of the model (Fig. 3E), which showed good utility. We also validated the model using the validation set of patients (n = 157). The ROC curve showed that the application value of the model was retained in the validation set (AUC = 0.814, 95% CI: 0.744–0.884; Fig. 3B). The efficacy of the model in the validation set was confirmed with a calibration plot (Fig. 3D) and decision curve (Fig. 3F).

### Comparison between the clinical-ultrasound model and individual models

**Fig. 4:**
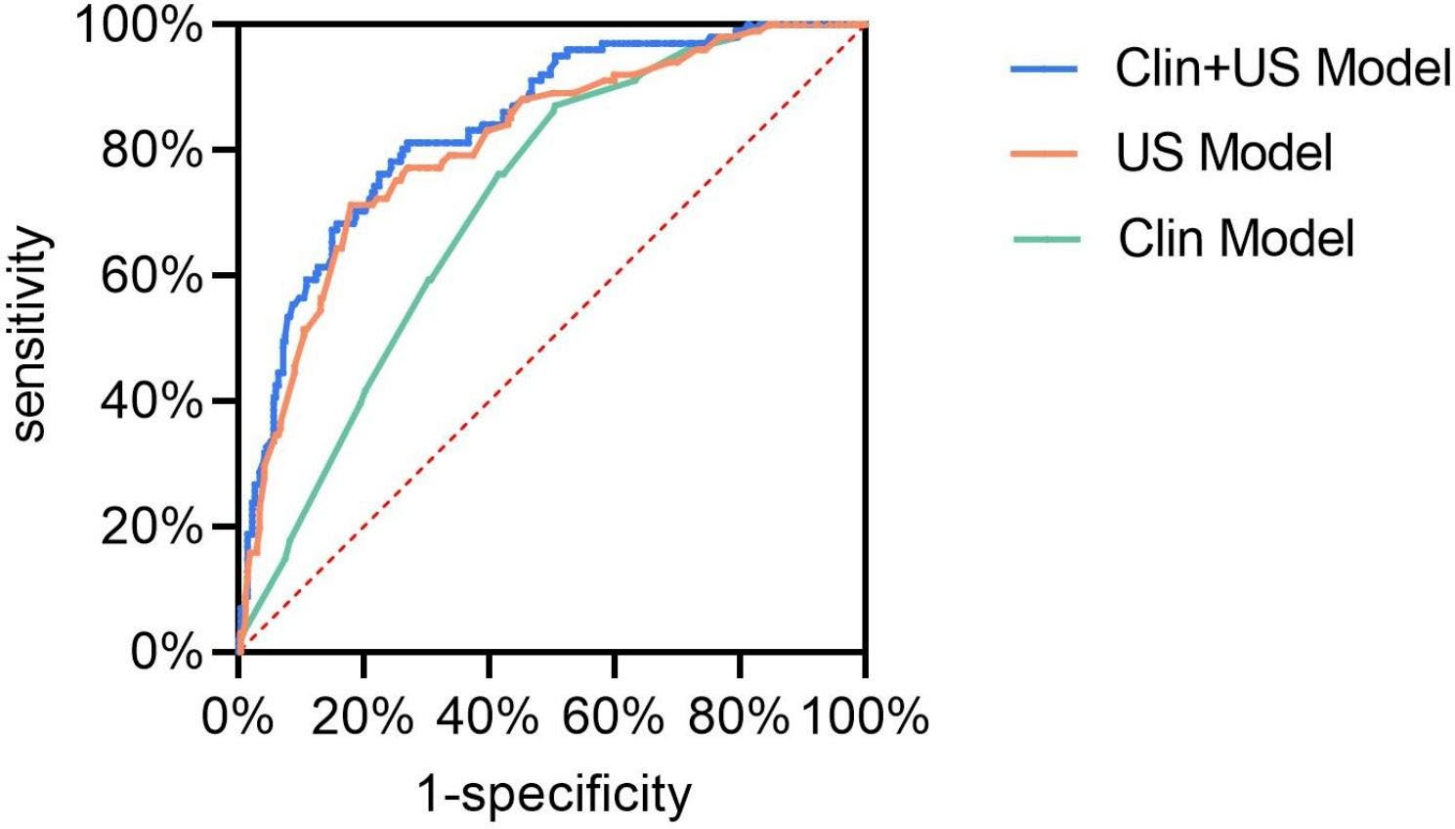
Comparison of combined model with clinical model and ultrasound model. Abbreviations: US Model, ultrasonic model; Clin Model, clinical model; Clin + US Model, clinical + ultrasonic model

We also compared our model with the individual clinical model and ultrasound model. The results showed that the model combining clinical and ultrasound data had a higher diagnostic value than either of the individual models (Fig. 4). Similarly, individual clinical and ultrasound nomograms were constructed using the same method as for the combined model. The clinical model (AUC = 0.715, 95% CI: 0.661–0.769) performed worse than the ultrasound model (AUC = 0.809, 95% CI: 0.759–0.858); both were inferior to the combined model.

## Discussion

In this multicenter study, we established a prediction model based on clinical and ultrasound data to evaluate the risk of lymph node metastasis in patients with ovarian cancer. Through univariate and multivariate regression analysis, we identified maximum tumor diameter, multifocal tumor, and Ki67 as independent risk factors for lymph node metastasis; these were included in the model. We evaluated the performance and clinical utility of the model using ROC curves, calibration plots, and decision curves and compared it with models using only clinical or ultrasound data. The results showed that the combined model had a greater diagnostic value than either of the individual models.

Ovarian cancer is a common gynecological malignancy, and surgical treatment is usually divided into radical and conservative surgery [11, 12]. In radical surgery, lymph node dissection is performed to treat and prevent ovarian cancer metastasis [13]. However, lymph node dissection also carries certain risks [14–16], such as postoperative lymphedema and lower limb deep vein thrombosis. Kleppe et al. [17] reported that the average incidence of lymph node metastasis in clinical stage I-II ovarian cancer was 14.2%. A separate study indicated that only 12.6% of patients with low-grade stage I-II ovarian cancer had undergone lymph node metastasis [18]. In our study, only 12 patients (11.9%) with stage I-II disease in the training set and 7 patients (14%) with stage I-II disease in the validation set had lymph node metastasis. Performing lymph node dissection on patients with a very low likelihood of lymph node metastasis may not be beneficial. Therefore, doctors need to evaluate the patient’s specific condition and surgical indications to decide whether to perform lymph node dissection. However, accurately identifying patients with true lymph node metastasis who would benefit from preoperative lymph node dissection is challenging. Predicting whether a patient will have lymph node metastasis before surgery is therefore particularly important.

Research on preoperative prediction of lymph node metastasis in ovarian cancer has so far failed to find a sufficiently accurate model. Most prediction models are based on clinical data, which are sometimes supplemented by CT or MRI. However, relevant literature indicates that CT and MRI cannot detect cancer in normal-sized lymph nodes, nor can they distinguish between hyperplastic and metastatic lymph nodes [19]. This has contributed to the poor accuracy of existing models in predicting lymph node metastasis. Ultrasound is widely used in the diagnosis of ovarian cancer as it is noninvasive and cost-effective. If ultrasound could be used to accurately predict lymph node metastasis, it would have significant clinical implications for patients. However, the combination of ultrasound data with other parameters for detecting lymph node metastasis is rare. In previous studies, age, CA125, Ki67, tumor diameter, histological type, histological grade, menopausal status, and other factors were shown to be associated with lymph node metastasis [20–25]. Our research findings were similar to this study; however, in our study, histological type was significant in the univariate but not in the multivariate analysis. We found that maximum tumor diameter, multifocal tumor, and Ki67 level were independent risk factors for lymph node metastasis. Furthermore, the FIGO and TNM stages were previously shown to be closely associated with lymph node metastasis [20, 25]. However, as these stages are evaluated based on lymph node status, we deemed it inappropriate to include them as study variables.

In our study, the maximum tumor diameter in the LNM negative group was significantly lower than that in the LNM positive group (81.67 ± 49.22 vs. 110.31 ± 49.02, *p* < 0.001). Multivariate analysis showed that tumor size was predictive of lymph node metastasis. As the tumor size increased, the probability of lymph node metastasis also increased. A previous large-scale retrospective analysis of 4,110 patients showed that when the tumor diameter was < 200 mm, the risk of lymph node metastasis increased with the tumor diameter [26], which is consistent with our results. However, this study also determined that the risk of lymph node metastasis in patients with a tumor diameter > 200 mm was lower than that in patients with a tumor diameter of 150 ~ < 200 mm. This anomaly may be due to the transformation of tumor diameter from measurement data to count data, leading to the loss of some statistical information. In addition, we found that the risk of lymph node metastasis was higher in multifocal than in single tumors. This is not surprising, as most multifocal tumors have higher invasiveness and malignancy than single tumors, and a greater likelihood of distant metastasis [27]. Moreover, this is an advantage of using ultrasound examination, as it can quickly identify multifocal tumors and determine the presence of suspicious metastatic lesions in the pelvis and abdomen. The expression level of Ki67 is widely used to predict the growth rate and potential malignancy of tumors [28]. Generally, a higher Ki67 level indicated increased tumor cell activity, leading to a faster rate of tumor growth and a greater potential for malignancy [29]. Our study results showed that Ki67 is a risk factor for lymph node metastasis, which is consistent with previous studies [3, 4].

We also established separate prediction models based on ultrasound examination or clinical data alone, and the results showed that the predictive power of the ultrasound examination-only model was superior to that of the clinical-only model, although both were inferior to the combined model. This highlighted the value of ultrasound examination in predicting lymph node metastasis in patients with ovarian cancer. The high AUC of the ultrasound model (AUC = 0.809, 95% CI: 0.759–0.858) indicated that the risk of lymph node metastasis in patients could be accurately predicted using noninvasive ultrasound examination. In addition, we used data from multiple medical centers, with a relatively large sample size. The prediction model based on ultrasound examination results and clinical data showed good accuracy in both the training and validation sets, indicating that our model has a good predictive value. Our study results can provide an important reference for the determination of lymph node metastasis in patients with ovarian cancer, which impacts the prognosis and treatment of patients. In our model, maximum tumor diameter, multifocal tumor, and Ki67 level are independent risk factors for lymph node metastasis. Therefore, when examining patients with ovarian cancer, these risk factors should be particularly noted to more accurately evaluate the patient’s risk of lymph node metastasis and develop the best individual treatment plan. The dynamic nomogram established in this study should facilitate the application of our model and thus help physicians make more accurate decisions. It should be noted that Ki67 was often obtained from immunohistochemistry of surgically resected tumor tissue. We recommend using immunohistochemical markers from preoperative biopsy specimens of ovarian cancer for preoperative prediction of LNM.

It should be noted that our study has some limitations. First, our study was based on retrospective data analysis, which may have information bias. Second, we conducted only internal and not external validation. Future studies are needed to further validate the nomogram in different populations.

## Conclusions

We developed a model for predicting lymph node metastasis in patients with ovarian cancer, utilizing ultrasound examination results and clinical data. In the current era of precision medicine, this model can aid in the more accurate evaluation of an individual’s risk of lymph node metastasis and therefore in determining the most suitable treatment plan for the patient.

### Electronic supplementary material

Below is the link to the electronic supplementary material.

Supplementary Material 1

## Data Availability

Data described in the manuscript will be made available upon request pending application and approval from the corresponding author.

## List of abbreviations

AUC: Area under the curve
CA: Cancer antigen
CI: Confidence interval
CT: Computed tomography
FIGO: International Federation of Gynecology and Obstetrics
LNM: Lymph node metastasis
MRI: Magnetic resonance imaging
OR: Odds ratio
ROC: Receiver operating characteristic
WBC: White blood cell
