# Supplementary material for: Nomogram for predicting lymph node metastasis in patients with ovarian cancer using ultrasonography: a multicenter retrospective study
Source: BMC Cancer. 2023 Nov 17;23:1121. doi: 10.1186/s12885-023-11624-5 (PMC10655276; doi:10.1186/s12885-023-11624-5)
Supplement: Supplementary file 1 — Supplementary Material 1 [file 12885_2023_11624_MOESM1_ESM.docx]

**Table S1** Baseline clinical data for all patients.

| Variables | All patients (N =525) | Training Set (N =368) | Validation Set (N =157) | P values |
| --- | --- | --- | --- | --- |
| Age (year) | 53.27±11.05 | 53.92±10.90 | 51.73±11.30 | 0.041 |
| Pregnancy history | 2.87±1.71 | 2.95±1.76 | 2.67±1.58 | 0.090 |
| Reproductive history | 1.59±1.03 | 1.59±1.02 | 1.59±1.07 | 0.976 |
| Menarche age (year) | 13.43±1.59 | 13.39±1.69 | 13.53±1.32 | 0.379 |
| Menopause | 355(67.62) | 245(66.58) | 110(70.06) | 0.434 |
| Irregular bleeding | 23(4.38) | 14(3.80) | 9(5.73) | 0.323 |
| Histological type |  |  |  | 0.119 |
| SOC | 338(64.38) | 234(63.59) | 104(66.24) |  |
| MOC | 29(5.52) | 15(4.08) | 14(8.92) |  |
| EEOC | 26(4.95) | 22(5.98) | 4(2.55) |  |
| OCCC | 112(21.33) | 80(21.74) | 32(20.38) |  |
| Others | 20(3.81) | 14(3.80) | 6(3.82) |  |
| FIGO stage |  |  |  | 0.051 |
| Ⅰ | 158(30.10) | 109(29.62) | 49(31.21) |  |
| Ⅱ | 77(14.67) | 55(14.95) | 22(14.01) |  |
| Ⅲ | 280(53.33) | 163(44.29) | 117(74.52) |  |
| Ⅳ | 61(11.62) | 41(11.14) | 20(12.74) |  |
| PR (+) | 150(28.57) | 111(30.16) | 39(24.84) | 0.216 |
| Ki67 (%) | 52.45±26.48 | 51.54±27.56 | 54.58±23.68 | 0.231 |
| CA125 (U/mL) | 846.83±1499.60 | 863.98±1504.24 | 807.21±1493.49 | 0.710 |
| CA153 (U/mL) | 58.87±89.54 | 54.78±80.75 | 67.66±106.13 | 0.314 |
| CA199 (U/mL) | 648.77±7120.38 | 812.87±8483.40 | 275.82±1530.09 | 0.471 |
| CA724 (IU/mL) | 41.33±100.44 | 46.76±113.23 | 29.77±64.59 | 0.244 |
| AFP (ng/mL) | 66.80±866.02 | 13.03±136.41 | 182.40±1522.75 | 0.106 |
| CEA (ng/mL) | 4.88±22.84 | 5.79±27.08 | 2.83±6.36 | 0.290 |
| SCC (ng/mL) | 0.81±0.86 | 0.82±0.99 | 0.79±0.51 | 0.807 |
| HE4 (pmol/L) | 290.69±336.62 | 279.93±326.42 | 315.27±360.49 | 0.499 |
| D-dimer (ug/mL) | 51.28±330.21 | 52.00±346.13 | 49.59±290.71 | 0.936 |
| RBC (x10^12^/L) | 3.69±0.63 | 3.68±0.63 | 3.72±0.64 | 0.454 |
| WBC (x10^9^/L) | 7.98±14.11 | 8.18±16.64 | 7.52±4.37 | 0.626 |
| Neutrophil (%) | 70.65±13.00 | 70.81±12.71 | 70.21±13.70 | 0.649 |
| Lymphocyte (%) | 20.08±9.96 | 20.04±9.86 | 20.18±10.23 | 0.886 |
| NLR | 5.60±6.41 | 5.61±6.57 | 5.55±6.04 | 0.916 |
| PLT (x10^9^/L) | 274.60±110.87 | 275.38±112.24 | 272.82±107.99 | 0.810 |

Abbreviations: SOC, serous ovarian carcinoma; MOC, mucinous ovarian carcinoma; EEOC, endometrioid ovarian carcinoma; OCCC, ovarian clear cell carcinoma; FIGO, international federation of gynecology and obstetrics; NLR, neutrophil-to-lymphocyte ratio. Data are presented as mean ± standard deviation or n (%).

**Table S2** Baseline ultrasound data from all patients.

| Variables | All patients (N =525) | Training Set (N =368) | Validation Set (N =157) | P values |
| --- | --- | --- | --- | --- |
| Maximum tumor diameter (mm) | 92.63±51.59 | 89.53±50.74 | 99.89±52.99 | 0.035 |
| Multifocal tumor | 224(42.67) | 158(42.93) | 66(42.04) | 0.849 |
| Laterality |  |  |  | 0.515 |
| Left | 176(33.52) | 128(34.78) | 48(30.57) |  |
| Right | 150(28.57) | 106(28.80) | 44(28.03) |  |
| Bilateral | 199(37.90) | 134(36.41) | 65(41.40) |  |
| Shape, circle | 265(50.48) | 189(51.36) | 76(48.41) | 0.536 |
| Homogeneous echoic | 405(77.14) | 284(77.17) | 121(77.07) | 0.979 |
| Calcification | 27(5.14) | 18(4.89) | 9(5.73) | 0.741 |
| Rear echo | 169(32.19) | 117(31.79) | 52(33.12) | 0.744 |
| UF | 186(35.43) | 131(35.60) | 55(35.03) | 0.901 |
| AM | 36(6.86) | 23(6.25) | 13(8.28) | 0.399 |
| Intimal thickness (mm) | 3.41±4.52 | 3.22±4.24 | 3.85±512 | 0.218 |

Abbreviations: UF, uterine fibroids; AM, adenomyosis

**Table S3** Comparison of CT and MR findings between the two groups of patients in the training set.

| Variables | LNM Negative (N =267) | LNM Positive (N =101) | P values |
| --- | --- | --- | --- |
| Maximum tumor diameter (mm) | 83.56±63.17 | 107.31±56.13 | <0.001 |
| Laterality |  |  | <0.001 |
| left | 105(39.32) | 22(21.78) |  |
| right | 84(31.46) | 21(20.79) |  |
| Bilateral | 78(29.21) | 58(57.43) |  |
| Shape, circle | 127(47.57) | 48(47.52) | 0.882 |
| FIGO staging by CT and MRI |  |  | <0.001 |
| Ⅰ | 113(42.32) | 6(5.94) |  |
| Ⅱ | 38(14.23) | 7(6.93) |  |
| Ⅲ | 91(34.08) | 60(59.41) |  |
| Ⅳ | 25(9.36) | 28(27.72) |  |

Data are presented as mean ± standard deviation or n (%).
